# Evidence of Cuticle Chemicals of *Heortia vitessoides* (Lepidoptera: Crambidae) Larvae Influencing the Aggregation Behavior of Conspecific Larvae

**DOI:** 10.3390/insects15100746

**Published:** 2024-09-26

**Authors:** Xinya Yang, Guangsheng Li, Cai Wang

**Affiliations:** College of Forestry and Landscape Architecture, South China Agricultural University, Guangzhou 510642, China; yxy@stu.scau.edu.cn (X.Y.); 20232159010@stu.scau.edu.cn (G.L.)

**Keywords:** aggregation preference, *Aquilaria sinensis*, extraction, gregariousness, Lepidoptera

## Abstract

**Simple Summary:**

*Heortia vitessoides* (Lepidoptera: Crambidae) larvae usually form large aggregates during young instars, but their gregariousness is reduced during the late instar. Our study shows that hexane extract of second-instar *H*. *vitessoides* larvae triggered the aggregation preference of the conspecific larvae, whereas the hexane extract of later-instar larvae was no longer attractive. In addition, acetone extracts of both second- and fifth-instar *H*. *vitessoides* larvae repelled the conspecific larvae.

**Abstract:**

*Heortia vitessoides* (Lepidoptera: Crambidae) is a severe pest of *Aquilaria* plants, which produce high-priced agarwood. The larval stage of this pest is gregarious, usually forming large aggregates during young instars and becoming solitary during the fifth instar. We hypothesize that the cuticle chemicals of young-instar *H. vitessoides* larvae could promote larval aggregating, whereas the cuticle chemicals of late-instar larvae would no longer attract young-instar larvae. In this study, two-choice tests were conducted to evaluate the effect of cuticle extracts of second- and fifth-instar *H. vitessoides* larvae on the aggregation preference of second-instar larvae. Results show that significantly more larvae aggregated on the leaves treated with the hexane extract of second-instar *H. vitessoides* larvae than on untreated leaves. However, the hexane extract of fifth-instar larvae had no significant effect on the aggregation preference of the second-instar conspecific larvae. Interestingly, acetone extracts of both second- and fifth-instar *H. vitessoides* larvae repelled the second-instar conspecific larvae throughout the 8 h experiment. Our study shows that cuticle chemicals of *H. vitessoides* larvae may play a role in the group dynamics of this pest, which may contribute to screening novel attractants and repellents for *H. vitessoides*. Detailed chemical analyses of the extracts and identification of the compounds involved in larval attracting and repelling would be valuable in future studies.

## 1. Introduction

*Heortia vitessoides* (Lepidoptera: Crambidae) is a severe pest of plants in the genus *Aquilaria*, which produces high-priced agarwood. In China, *H. vitessoides* mainly damages *Aquilaria sinensis* and poses great threats to the agarwood industry in two aspects. Firstly, *H. vitessoides* can consume large amounts of *A. sinensis* leaves in a short period, causing weakness and even death of the tree. Secondly, farmers usually need to apply a large amount of pesticides to control *H. vitessoides* in *A. sinensis* plantations multiple times each year, resulting in pesticide residue problems that greatly reduce the quality and price of agarwood products. Xu et al. [1] estimated that the suitable distribution area of *H. vitessoides* could reach >1.3 × 10^6^ km^2^ in China, indicating an increase in economic losses caused by this pest in the near future. Recently, an increased number of studies have focused on the behavior, physiology, chemical ecology, and molecular biology of *H. vitessoides* [2,3,4,5], which may contribute to effectively controlling this pest and reducing the use of pesticides.

An interesting characteristic of *H. vitessoides* is the aggregation behavior of this pest during the larval stage. Our previous study shows that *H. vitessoides* larvae can form large aggregates before the third instar, with a single cohort containing several dozens or hundreds of individuals [6]. Interestingly, we observed the merging of young-instar cohorts that form larger aggregates under field and laboratory conditions. In addition, both newly hatched and second-instar larvae show a strong tendency to reaggregate after being separated. However, the group sizes of *H. vitessoides* larvae sharply decreased after the third instar, and they became solitary during the fifth instar. The aggregation behaviors of larvae may present a novel target for *H. vitessoides* control. For example, Liang et al. [6] reported that pesticides such as avermectin can be horizontally transferred from larvae directly exposed to the pesticide to unexposed cohort members through body contact. In addition, Qian et al. [7] reported the transmission of *Metarhizium anisopliae* within *H. vitessoides* cohorts.

However, the mechanism behind the aggregation behaviors of *H. vitessoides* larvae is unclear. We hypothesized that young-instar *H. vitessoides* larvae could produce chemicals to attract conspecific larvae and maintain the aggregation. Since the aggregation behavior reduced through development, we also hypothesized that older *H. vitessoides* larvae would no longer attract young-instar larvae. Here, we first obtained hexane or acetone extract of second-instar *H. vitessoides* cohorts or solitary fifth-instar larvae. The two-choice tests were then conducted to investigate the aggregation preferences of second-instar *H. vitessoides* larvae to leaves treated with cuticle extract versus untreated leaves.

## 2. Materials and Methods

### 2.1. Insects

Methods provided by Liang et al. [6] were used to collect *H. vitessoides* larvae from an *A. sinensis* plantation located in Tianlu Lake Park (23°15′ N, 113°24′ E), Huangpu, Guangzhou, China. No pesticide was applied in this plantation for >6 months before and during larvae collection. The leaves and branches damaged by *H. vitessoides* larvae were carefully searched and cut using averruncator shears. A cohort was determined if many *H. vitessoides* larvae (with body contact) were found in the same leaf or branch. The instar of the larvae was determined using the method described by Qiao et al. [8]. Three cohorts composed of second-instar larvae were randomly collected from different *A. sinensis* trees, and each cohort was placed in a 4800 mL plastic container (top diameter: 29 cm; bottom diameter: 23.5 cm; height: 9 cm) with fresh *A. sinensis* leaves (Table 1). We also collected six fifth-instar larvae (which had become solitary) from different trees, and each larva was placed in a container. These larvae were brought to the laboratory within 2 h for cuticle chemical extraction. In addition, several cohorts of second-instar larvae were collected as described above to set the two-choice tests. These larvae were reared in the laboratory at 26 ± 2 °C and under a 12:12 light–dark schedule, with fresh *A. sinensis* leaves provided each day.

### 2.2. Extraction Preparation

Cuticle chemicals of *H. vitessoides* larvae were extracted using hexane (HPLC grade, 95%, Thermo Fisher Scientific, Cleveland, OH, USA) and acetone (analytical grade, 99.5%, Guangzhou Chemical Co., Guangzhou, China). For each solvent, 200–300 mg of second-instar larvae from each of the three cohorts were randomly selected and weighed using a 0.1 mg electronic balance (Table 1). Each fifth-instar larva (ranging from 200–300 mg for each larva) was also weighed. Each sample was placed in a Petri dish (diameter: 9 cm), and 2 mL distilled water was added. The larvae (larva) were immersed in distilled water for 30 sec and transferred onto a Whatman filter paper (No. 1, Whatman International Ltd., Maidstone, UK). These larvae were allowed to walk on the filter paper for 2 min to dry and then transferred to a 10 mL graduated test tube. The solvent (hexane or acetone) was added into the tube at a ratio of 100 mg larvae (larva) to 3 mL solvent, and larvae were soaked in the solvent for 10 min. The extracts were filtered using a 0.45 μm microporous membrane and concentrated by blowing nitrogen gas until the solvent volume decreased to 66%. In total, 12 extract samples (i.e., three hexane or acetone extracts of cuticle chemicals of second- or fifth-instar *H. vitessoides* larvae) were obtained and stored in a −80 °C freezer.

### 2.3. Aggregation-Choice Test

**Figure 1:**
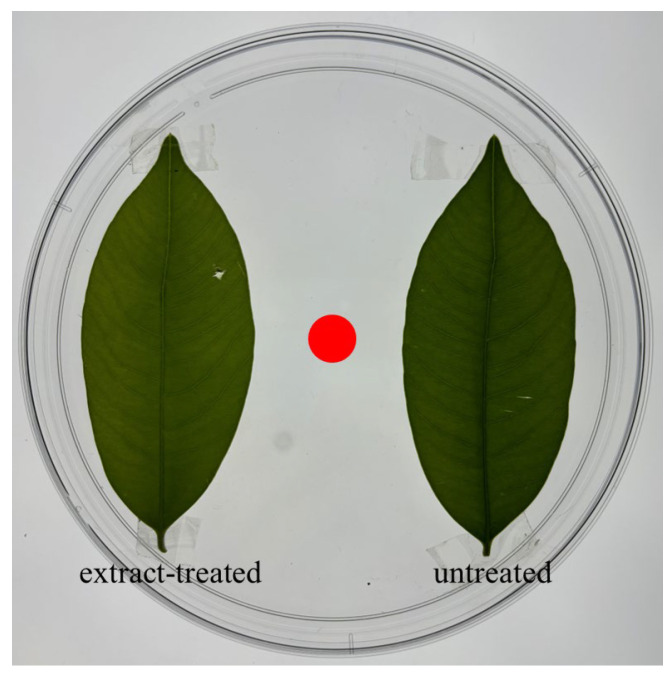
In each aggregation choice test, extract-treated and untreated leaf was pasted on the two sides of the bottom of a Petri dish. Ten second-instar *H. vitessoides* larvae were release on the center of the Petri dish (indicated by the red dot) at the beginning of the experiment.

Methods provided by Qian et al. [9] were slightly modified to investigate the effect of hexane or acetone extracts of cuticle chemicals on the aggregation preference of second-instar *H. vitessoides* larvae. In brief, two *A. sinensis* leaves (collected from the same branch) were washed using distilled water and dried. One hundred microliters of extract (i.e., obtained from 5 mg of larvae/larva) was added onto the surface of one leaf and evenly smeared. The same amount of non-extracted solvent (hexane or acetone) was added to another leaf. These leaves were placed on the lab table for 10 min until the hexane or acetone completely evaporated. The extract-treated and untreated leaf was then pasted on the two sides of the bottom of a Petri dish (diameter: 15 cm; height: 2.5 cm) (Figure 1). Ten second-instar *H. vitessoides* larvae were randomly selected from the same cohort and placed onto the center of the bottom side of the Petri dish, and a lid was placed on it. In total, there were 120 experiment units, with ten replicates for each extract mentioned above. The cardinal direction of each Petri dish was randomly assigned. The bioassays were maintained at 26 ± 2 °C. At each hour until 8 h, the number of *H. vitessoides* larvae that stayed on the extract-treated or untreated leaf or on the surface of the Petri dish was counted.

### 2.4. Data Analysis

In each two-choice test, the percentage of *H. vitessoides* larvae found in each location (treated leaves, untreated leaves, and the Petri dish) was calculated. The log-ratio transformation was used to make the percentage data independent [10], with 0 being replaced with 5%, which is half of the smallest value recorded in this test [11]. For each extract (hexane or acetone) and each development stage (second or fifth instar), the transformed data were analyzed using two-way analysis of variance (ANOVA) with the cohorts (used for extraction) as a random effect and location as a fixed effect, followed by Tukey’s HSD test for multiple comparisons (SAS 9.4, SAS Institute, Cary, NC, USA). The attractive effect of the extract was determined if significantly more *H. vitessoides* larvae aggregated on the extract-treated leaves than on untreated leaves. On the contrary, the repelling effect of the extract was determined if significantly fewer larvae were found on the extract-treated leaves than on untreated leaves.

## 3. Results

### 3.1. Effect of Cuticle Extract of Second-Instar Larvae on Aggregation Preference of Heortia vitessoides Larvae

Significantly more larvae aggregated on the leaves treated with the hexane extract of cuticle chemicals of second-instar larvae than untreated leaves at 1, 2, 4, and 5 h (Table 2). However, significantly fewer larvae were found on the leaves treated with the acetone extract of cuticle chemicals of second-instar larvae than untreated leaves throughout the experiment, except for at 7 h (Table 3).

### 3.2. Effect of Cuticle Extract of Fifth-Instar Larvae on Aggregation Preference of Heortia vitessoides Larvae

There was no significant difference in the percentage of larvae found on the leaves treated with the hexane extract of cuticle chemicals of fifth-instar larvae and untreated leaves (Table 4). Significantly fewer larvae were found on the leaves treated with the acetone extract of cuticle chemicals of fifth-instar larvae than on untreated leaves throughout the 8 h experiment (Table 5).

## 4. Discussion

In conclusion, our study shows that hexane extract of cuticle chemicals of second-instar *H. vitessoides* larvae significantly triggered the aggregation behavior of conspecific larvae, whereas hexane extract of fifth-instar larvae did not affect the aggregation preferences of conspecific larvae. These results support our hypothesis that early-instar *H. vitessoides* larvae produce cuticle chemicals attracting conspecific larvae. However, acetone extract of both second- and fifth-instar larvae had repelling effects on *H. vitessoides* larvae.

It is unclear which compounds in the hexane extract of second-instar larvae triggered the aggregation preference of the conspecific larvae. Although many studies have reported aggregation behaviors of lepidopteran larvae, very few of them have identified the semiochemicals that contribute to the aggregation [7]. For example, Kwadha et al. [12] reported that *Galleria mellonella* produce decanal to attract conspecific larvae. In addition, *Cydia pomonella* uses a series of volatiles including (E)-2-octenal, geranyl acetone, (E)-2-nonenal, and sulcatone to recruit pupating larvae [13]. Some cuticular hydrocarbons may also play roles in nestmate recognition and communication of group-living insects [14,15,16]. It would be interesting to identify chemicals in the hexane extracts that attract *H. vitessoides* larvae. The hexane would extract diverse non-polar chemicals, including small molecules and long-chain cuticular hydrocarbons, and the identification of these chemicals would be challenging. In a recent study, Du et al. [17] fractionated ylang ylang oil using a flash chromatography system and obtained 70 fractions. By testing the behavioral responses of *Solenopsis invicta* to each chromatographic fraction using two-choice tests, they eventually screened and identified two compounds that attract alates and workers. Such a strategy might be useful to identify compounds in the hexane extract of second-instar *H. vitessoides* that attract conspecific larvae.

Since horizontal transfer of pesticides and entomopathogenic fungi within *H. vitessoides* cohorts has been reported [6,9], applying *H. vitessoides* attractants may enhance larval aggregation and increase the effectiveness of pesticides or biocontrol agents. However, the aggregation effect triggered by the hexane extracts of second-instar larvae wore off with time (Table 2). This may be due to two possible reasons: (1) after the treated leaves are eaten by larvae, some of them may move to the untreated leaves; (2) the amount of attracting chemicals may decrease through time because of evaporation or decomposition. For the latter situation, the aggregation effects of attractants may not last for a significant duration under natural conditions, therefore limiting their application.

The aggregation behavior plays an important role in the development of *H. vitessoides* during early instars. For example, Liang et al. [6] reported that a newly hatched larva isolated from the cohort stopped feeding, continued wandering to search for the conspecific larvae, and eventually died. Huang et al. [18] also reported that *H. vitessoides* individuals living in larger cohorts (n = 90) had significantly larger body sizes and developed faster than those that lived in small cohorts (n = 30). However, overly large cohorts and the extremely high density of group-living larvae may result in strong intraspecific competition to limited food sources and draw the attention of predators [19,20,21]. Therefore, a common strategy applied by many gregarious species is to maintain moderate group sizes [7]. In addition, the aggregation behaviors could be dynamic, with group sizes decreasing with the development of larvae to reduce competition [22,23,24]. In our study, the cuticle compounds (hexane extract) of fifth-instar *H. vitessoides* larvae no longer attracted conspecific larvae. Comparing the differentially produced chemicals among second- and fifth-instar larvae may also contribute to screening compounds that would trigger the aggregation behavior of *H. vitessoides* larvae.

Interestingly, we found that acetone extracts of both second- and fifth-instar larvae repelled conspecific larvae. This might be the first evidence that gregarious lepidopteran larvae could produce chemicals to adjust the level of gregariousness by reaching a balance of attracting and repelling cohort members. Acetone extracts more polar compounds but also extracts significant amounts of the non-polar compounds. It would be valuable to screen repellent chemicals from acetone extracts, which may contribute to protecting *A. sinensis* trees from *H. vitessoides* attacks.

## Figures and Tables

**Table 1:** Basic information of *Heortia vitessoides* cohorts (second instar) and individuals (fifth instar) collected and used for cuticle extraction.

Instar	Cohort (2nd Instar)/ Individual (5th Instar)	Collection Date (yy/mm/dd)	Group Number/Cohort	Weight of Larvae Used for Extraction (mg)	
				Hexane	Acetone
2nd	1	7 May 2024	99	233.1	236.3
	2	7 May 2024	184	257.7	253.3
	3	7 May 2024	275	232.6	229.4
5th	1	15 May 2024	1	278.1	-
	2	15 May 2024	1	267.0	-
	3	15 May 2024	1	276.2	-
	4	15 May 2024	1	-	269.4
	5	30 May 2024	1	-	268.7
	6	30 May 2024	1	-	273.8

**Table 2:** Percentage (mean ± SE) of *Heortia vitessoides* larvae aggregated on the leaves treated with the hexane extract of cuticle chemicals of second-instar *Heortia vitessoides* larvae or untreated leaves or the Petri dish. Different letters within the same row indicate significant differences (*p* < 0.05).

Time Point	Treated Leaf	Control Leaf	Petri Dish	Statistical Results
1 h	52.3 ± 7.1 a	32.3 ± 5.7 b	15.3 ± 5.2 c	*F* = 32.87; df = 2,81; *p* < 0.0001
2 h	53.0 ± 4.3 a	33.3 ± 3.8 b	13.7 ± 3.1 c	*F* = 35.33; df = 2,81; *p* < 0.0001
3 h	52.7 ± 4.5 a	36.3 ± 4.2 a	11.0 ± 2.5 b	*F* = 37.05; df = 2,81; *p* < 0.0001
4 h	52.7 ± 4.8 a	33.0 ± 4.3 b	14.3 ± 3.1 c	*F* = 21.44; df = 2,81; *p* < 0.0001
5 h	48.0 ± 4.8 a	29.0 ± 4.3 b	23.0 ± 3.6 b	*F* = 7.94; df = 2,81; *p* = 0.0007
6 h	39.7 ± 5.3 a	28.7 ± 3.9 a	31.7 ± 4.5 a	*F* = 0.86; df = 2,81; *p* = 0.4271
7 h	31.6 ± 4.7 a	28.7 ± 3.6 a	39.7 ± 4.7 a	*F* = 1.24; df = 2,81; *p* = 0.2942
8 h	32.6 ± 4.9 a	28.4 ± 4.6 a	39.0 ± 4.0 a	*F* = 2.61; df = 2,81; *p* = 0.0797

**Table 3:** Percentage (mean ± SE) of *Heortia vitessoides* larvae aggregated on the leaves treated with the acetone extract of cuticle chemicals of second-instar *Heortia vitessoides* larvae or untreated leaves or the Petri dish. Different letters within the same row indicate significant differences (*p* < 0.05).

Time Point	Treated Leaf	Control Leaf	Petri Dish	Statistical Results
1 h	27.7 ± 3.5 b	48.0 ± 4.1 a	24.3 ± 3.1 b	*F* = 10.07; df = 2,81; *p* = 0.0001
2 h	28.3 ± 3.4 b	48.3 ± 3.8 a	23.3 ± 3.0 b	*F* = 13.31; df = 2,81; *p* < 0.0001
3 h	25.3 ± 3.8 b	47.3 ± 4.1 a	27.3 ± 3.4 b	*F* = 9.99; df = 2,81; *p* = 0.0001
4 h	25.3 ± 3.7 b	46.3 ± 5.0 a	28.7 ± 3.9 b	*F* = 6.26; df = 2,81; *p* = 0.0030
5 h	22.0 ± 4.1 b	39.0 ± 5.0 a	39.0 ± 4.7 a	*F* = 6.14; df = 2,81; *p* = 0.0033
6 h	17.3 ± 3.2 b	38.7 ± 4.4 a	44.0 ± 4.3 a	*F* = 16.75; df = 2,81; *p* < 0.0001
7 h	23.0 ± 3.7 b	35.7 ± 5.0 ab	41.0 ± 4.3 a	*F* = 6.38; df = 2,81; *p* = 0.0027
8 h	19.3 ± 4.1 b	40.7 ± 4.8 a	40.0 ± 4.4 a	*F* = 10.71; df = 2,81; *p* < 0.0001

**Table 4:** Percentage (mean ± SE) of *Heortia vitessoides* larvae aggregated on the leaves treated with the hexane extract of cuticle chemicals of fifth-instar *Heortia vitessoides* larvae or untreated leaves or the Petri dish. Different letters within the same row indicate significant differences (*p* < 0.05).

Time Point	Treated Leaf	Control Leaf	Petri Dish	Statistical Results
1 h	42.3 ± 3.7 a	38.0 ± 4.3 a	19.7 ± 2.7 b	*F* = 10.72; df = 2,81; *p* < 0.0001
2 h	44.7 ± 5.0 a	45.3 ± 5.1 a	10.0 ± 2.0 b	*F* = 25.75; df = 2,81; *p* < 0.0001
3 h	46.0 ± 5.4 a	46.3 ± 5.3 a	7.7 ± 1.8 b	*F* = 34.40; df = 2,81; *p* < 0.0001
4 h	44.0 ± 5.6 a	48.0 ± 5.4 a	8.3 ± 1.7 b	*F* = 30.75; df = 2,81; *p* < 0.0001
5 h	39.3 ± 5.3 a	46.0 ± 5.2 a	14.7 ± 2.9 b	*F* = 13.30; df = 2,81; *p* < 0.0001
6 h	39.0 ± 5.5 a	42.7 ± 5.6 a	18.3 ± 3.9 b	*F* = 7.22; df = 2,81; *p* = 0.0013
7 h	35.0 ± 5.7 a	35.7 ± 5.9 a	29.3 ± 6.0 a	*F* = 0.95; df = 2,81; *p* = 0.3901
8 h	26.7 ± 4.6 a	35.7 ± 5.3 a	37.7 ± 5.0 a	*F* = 1.58; df = 2,81; *p* = 0.2118

**Table 5:** Percentage (mean ± SE) of *Heortia vitessoides* larvae aggregated on the leaves treated with the acetone extract of cuticle chemicals of fifth-instar *Heortia vitessoides* larvae or untreated leaves or the Petri dish. Different letters within the same row indicate significant differences (*p* < 0.05).

Time Point	Treated Leaf	Control Leaf	Petri Dish	Statistical Results
1 h	21.0 ± 3.5 b	43.3 ± 4.7 a	35.7 ± 4.2 a	*F* = 9.77; df = 2,81; *p* = 0.0002
2 h	26.3 ± 4.5 b	51.0 ± 4.7 a	22.7 ± 3.0 b	*F* = 11.82; df = 2,81; *p* < 0.0001
3 h	28.3 ± 5.0 b	53.3 ± 5.1 a	18.3 ± 3.4 b	*F* = 13.46; df = 2,81; *p* < 0.0001
4 h	28.7 ± 5.2 b	56.0 ± 5.2 a	15.3 ± 3.2 b	*F* = 17.30; df = 2,81; *p* < 0.0001
5 h	27.7 ± 5.3 b	56.7 ± 5.1 a	15.7 ± 2.9 b	*F* = 18.36; df = 2,81; *p* < 0.0001
6 h	30.0 ± 5.3 b	56.0 ± 5.1 a	14.0 ± 2.6 c	*F* = 20.02; df = 2,81; *p* < 0.0001
7 h	28.3 ± 5.3 b	53.7 ± 5.7 a	18.3 ± 3.2 b	*F* = 12.60; df = 2,81; *p* < 0.0001
8 h	26.7 ± 4.6 b	56.0 ± 5.6 a	17.3 ± 3.1 b	*F* = 19.57; df = 2,81; *p* < 0.0001

## Data Availability

The raw data and materials will be made available by the authors, without undue reservation, to any qualified researchers.
